# Session Rating of Perceived Exertion Is a Superior Method to Monitor Internal Training Loads of Functional Fitness Training Sessions Performed at Different Intensities When Compared to Training Impulse

**DOI:** 10.3389/fphys.2020.00919

**Published:** 2020-08-12

**Authors:** Joao Henrique Falk Neto, Ramires Alsamir Tibana, Nuno Manuel Frade de Sousa, Jonato Prestes, Fabricio Azevedo Voltarelli, Michael D. Kennedy

**Affiliations:** ^1^Athlete Health Lab, Faculty of Kinesiology, Sport, and Recreation, University of Alberta, Edmonton, AB, Canada; ^2^Graduate Program on Physical Education, Catholic University of Brasilia, Brasilia, Brazil; ^3^Graduate Program in Health Sciences, Faculty of Medicine, Federal University of Mato Grosso (UFTM), Cuiabá, Brazil; ^4^Laboratory of Exercise Physiology, Faculty Estacio of Vitoria, Vitoria, Brazil

**Keywords:** resistance exercise, exercise training, functional performance, internal training load, high-intensity functional training

## Abstract

Despite its increase in popularity, little is known about how to best quantify internal training loads from functional fitness training (FFT) sessions. The purpose of this study was to assess which method [training impulse (TRIMP) or session rating of perceived exertion (sRPE)] is more accurate to monitor training loads in FFT. Eight trained males (age 28.1 ± 6.0 years) performed an ALL-OUT FFT session and an intensity-controlled session (RPE of six out of 10). Internal load was determined *via* Edward’s TRIMP (eTRIMP), Bannister’s TRIMP (bTRIMP), and sRPE. Heart rate was measured continuously during the session, while blood lactate and rate of perceived exertion were measured at baseline, and immediately and 30 min after the sessions. ALL-OUT blood lactate and RPE were significantly higher immediately and 30 min after the session compared to the RPE6 condition. ALL-OUT training load was significantly different between conditions using bTRIMP (61.1 ± 10.6 vs. 55.7 ± 12.4 AU) and sRPE (91.7 ± 30.4 vs. 42.6 ± 14.9 AU), with sRPE being more sensitive to such differences [*p* = 0.045, effect size (ES) = 0.76 and *p* = 0.002, ES = 1.82, respectively]. No differences in the training loads of the different sessions were found using eTRIMP (93.1 ± 9.5 vs. 84.9 ± 13.7 AU, *p* = 0.085). Only sRPE showed a significant correlation with lactate 30 min post session (*p* = 0.015; *p* = 0.596, large). sRPE was more accurate than both TRIMP methods to represent the overall training load of the FFT sessions. While the use of sRPE is advised, further research is necessary to establish its ability to reflect changes in fitness, fatigue, and performance during a period of training.

## Introduction

Functional fitness training (FFT) involves the performance of functional exercises (those that involve whole body, universal motor recruitment patterns, and executed in multiple planes of movement), in sessions that are short, intense, and that challenges multiple physiological systems at the same time (Feito et al., 2018; Falk Neto and Kennedy, 2019). Participants usually perform 3–5 whole body training sessions per week, with each session characterized as gymnastics, weightlifting, or metabolic, depending on which fitness components are being targeted (Drake et al., 2017; Crawford et al., 2018).

The structure of the metabolic conditioning sessions in FFT can vary significantly. The sessions are usually performed in a circuit, utilizing a combination of weightlifting and calisthenic exercises, often combined with short intervals of high-intensity cardiovascular work. Many of these sessions are performed as all-out efforts (Derek et al., 2018), where the goal is to complete the task in the shortest amount of time possible or to complete the highest amount of work in a set period of time (Crawford et al., 2018; Falk Neto and Kennedy, 2019; Tibana et al., 2019a). Not surprising, it has been shown that a single session of FFT can lead to high levels of oxidative, metabolic, cardiovascular, muscular, and immunological stress (Kliszczewicz et al., 2015; Tibana et al., 2016; Mate-Munoz et al., 2017; Kliszczewicz et al., 2018), with recovery from a FFT session requiring up to 48 h (Fernández-Fernández et al., 2015). As some component of metabolic training is performed almost daily on FFT programs (Crawford et al., 2018), constant bouts of all-out exercise can lead to excessive fatigue and ill health. A recent study showed that five FFT sessions per week over 4 weeks led to symptoms of functional overreaching (Drake et al., 2017), with non-functional overreaching likely to occur with continued stimulus. Therefore, long-term adherence to a FFT program may cause excessive fatigue and non-functional overreaching due to the all-out intensities constantly required in FFT sessions.

One way to ensure that this does not occur during a training program is by monitoring the training loads (Halson, 2014). The determination of training loads can be done by measuring the external (an objective measure of total work performed) and the internal load (the relative biological stress that is imposed on the system during training or competition; Bourdon et al., 2017). It has been suggested that a combination of internal and external loads is optimal to inform practitioners of changes in performance, health, and fatigue (Halson, 2014; Bourdon et al., 2017). However, given the different types of training sessions and the constant variability of FFT programs, the quantification of external training loads in FFT is a challenge (Derek et al., 2018). In this context, assessing the internal training loads in FFT would provide practitioners with insights about the physiological strain that each session imposes on participants. Such practice would likely reduce the chances of training load errors, which can increase the risk of injuries and non-functional overreaching (Halson, 2014; Bourdon et al., 2017).

Two commonly used methods to monitor training loads are the session rating of perceived exertion (sRPE) and the training impulse (TRIMP). The sRPE method has been proposed by Foster (1998) and considers the overall effort of the training session. When calculating the sRPE, the Borg-RPE (6–20) scale can apparently be used interchangeably with the Borg CR-10 scale (Arney et al., 2019). Recent evidence also suggests that sRPE not only provides information related to intensity, but also conveys information about progressive fatigue (Fusco et al., 2020a). In particular, sRPE provides information on accumulated fatigue that is not available from accepted markers of internal training intensity, such as heart rate and lactate concentrations. A previous study (Fusco et al., 2020a) suggested that sRPE progressively increased during a course of prolonged exercise training (within days) although objective measures of intensity, such as pace, heart rate, and lactate concentrations did not change, which was also noted by Foster et al. (2001). Recent evidence (Fusco et al., 2020b) also supports the concept that sRPE is a sensitive tool that may detect accumulated fatigue across multiple training days, in addition to being a surrogate marker of exercise intensity. In this context, in order to avoid inadequate recovery or overtraining, sRPE may be used to provide insight into accumulated fatigue during periods of increased training (Fusco et al., 2020b). On the other hand, the TRIMP method is commonly used for monitoring of aerobic training and was originally calculated based on training duration and changes in heart rate during the exercise session (Halson, 2014). Derivations of the original TRIMP method based on heart rate zones or individual thresholds have also demonstrated their validity in assessing the dose-response relationship to training (Edwards, 1993; Halson, 2014; Sanders et al., 2017).

Currently, it is unknown which method (sRPE or TRIMP) is more suitable for monitoring training loads of FFT sessions. The use of sRPE has shown promise as a valid method to determine the internal training load of FFT sessions (Derek et al., 2018; Tibana et al., 2018). However, despite being used to monitor training loads in a FFT athlete throughout a full season of training and competition (Tibana et al., 2019b), sRPE might still be prone to errors, particularly in the early stages of its utilization (Derek et al., 2018). While recent studies have found a strong correlation between Edward’s TRIMP (eTRIMP) and sRPE (Derek et al., 2018; Tibana et al., 2018), the protocols in these studies called for the typical all-out efforts during the sessions. Thus, the accuracy of these methods to determine the internal training load of FFT sessions performed at different intensities has not yet been examined.

Therefore, the primary aim of this study was to assess which method [Bannister’s TRIMP (bTRIMP), Edward’s TRIMP (eTRIMP), or sRPE] is more accurate to determine the training loads of FFT sessions performed at different intensities. As sRPE has been previously shown to be a valid measure of internal training load in FFT (Derek et al., 2018; Tibana et al., 2018), the relation between both TRIMP methods and sRPE is also analyzed. Secondly, we aimed to examine the relationship between blood lactate with both TRIMP methods and sRPE. The relationship between both TRIMP methods and RPE was also examined. It was hypothesized that the all-out session would lead to more time spent at intense heart rate training zones. This would lead to higher TRIMP scores that would be directly related with lactate levels and perceived exertion. Lastly, given the fact that the eTRIMP is based on five heart rate zones, it was hypothesized that it would more accurately reflect the cardiovascular response to the FFT sessions and, thus, be more accurate than bTRIMP for the calculation of internal training load.

## Materials and Methods

### Subjects

Eight males (age 28.1 ± 5.4 years, 23–39 years old) were recruited. Their anthropometric and performance characteristics are presented in Table 1. All participants were free of injury or known illnesses, were not using performance enhancing drugs, and had a minimum of 6 months of FFT experience (3.8 ± 1.4 years, 1.5–6 years of experience). Participants were advised to sleep at least 6–8 h the night before, maintain regular nutritional and hydration habits, avoid intense exercise 48 h prior to the sessions and to avoid smoking, alcohol, or caffeine consumption 24 h before a session. All participants provided informed consent, and the study was approved by the University Research Ethics Committee for Human Use (2.698.225/Universidade Estácio de Sá/UNESA/RJ) and conformed to the Declaration of Helsinki on the use of human participants for research.

**Table 1:** Participants’ characteristics (mean ± SD).

Body weight (kg)	77.18 ± 4.41
VO_2max_ (ml kg min^−1^)	52.10 ± 4.82
2000 m Rowing test (min)	7.35 ± 0.18
Mean power (WM)	239.88 ± 29.12
1 RM Back squat (kg)	135.57 ± 21.89
Back squat relative strength (1RM/BW)	1.74 ± 0.22
1 RM Front squat (kg)	123.14 ± 18.43
Front squat relative strength (1RM/BW)	1.58 ± 0.19
1 RM Snatch (kg)	79.71 ± 11.66
Snatch relative strength (1RM/BW)	1.02 ± 0.10
1RM Clean and jerk	102.42 ± 12.71
Clean and jerk relative strength (1RM/BW)	1.31 ± 0.11

### Study Design

**Figure 1:**
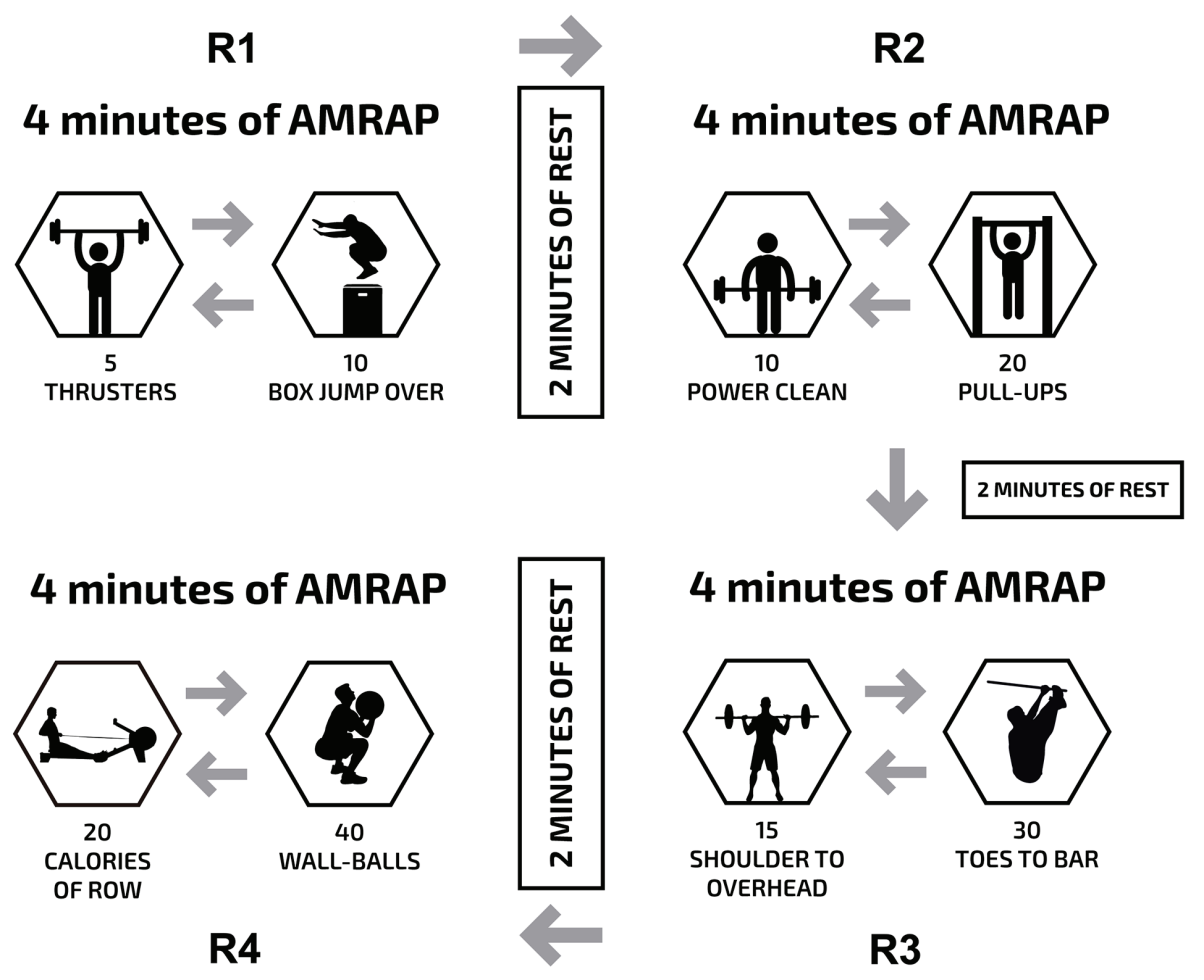
Description of the metabolic conditioning sessions (Tibana Test). AMRAP, as many rounds as possible.

The participants completed a metabolic conditioning training session (Figure 1; 5–7 days apart) in a randomized fashion under two different conditions: (a) all-out (ALL) and (b) intensity controlled (RPE6). The metabolic conditioning training session was the Tibana Test, which involved the completion of four different rounds of work, each separated by 2 min of rest (Figure 1). The rounds consisted of 4 min of as many rounds as possible (AMRAP) of five thrusters (60 kg) and 10 box jumps over (round 1); 4 min of AMRAP of 10 power clean (60 kg) and 20 pull-ups (round 2); 4 min of AMRAP of 15 shoulder to overhead (60 kg) and 30 toes to bar (round 3); and 4 min of AMRAP of 20 calories of rowing and 40 wall ball (9 kg; round 4).

**Figure 2:**
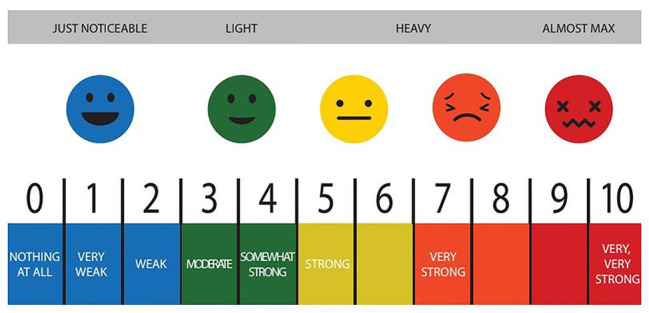
Rate of perceived exertion table made available to participants during the metabolic conditioning sessions.

During the all-out session, participants were instructed to complete the maximum number of repetitions possible for each round. In the RPE6 session, they performed the same conditioning session, but were told to self-regulate the intensity of their effort based on a perception of effort of six (hard) out of 10 on an adapted version of the Borg CR-10 scale (Foster et al., 2001; Morishita et al., 2018). In order to achieve this, participants were instructed to take more breaks if needed or to pace themselves in the execution of their exercises to keep the perception of effort at the desired level. No changes to the weights were performed during the sessions. The adapted Borg CR-10 scale was printed and available to the participants as a visual reminder of the prescribed target intensity (Figure 2). During each session, blood lactate, heart rate, and perceived exertion were collected.

### Heart Rate

Heart rate was continuously monitored during each session with the use of a Polar H10-HR monitor (Polar Electro Oy, Kemple, Finland). Participants’ maximal heart rate was obtained following a 2-km rowing test, utilized to indirectly assess the participants’ maximal oxygen uptake (VO_2max_; Klusiewicz et al., 2016). The test consists of rowing 2 km with the maximal effort (power) possible, aiming to complete the distance in the shortest time. Heart rate was continuously monitored during the test and the maximum HR observed during the test was used as the participants’ maximum heart rate. Maximal heart rate for every subject at the end of the test exceeded 90% of their age predicted maximum heart rate (HRmax = 220–0.64^*^age; Nes et al., 2013), indicating that a maximal effort was achieved.

### Blood Lactate

Capillary blood samples were collected through a transcutaneous puncture on the medial side of the tip of the middle finger using a disposable hypodermic lancet. Blood lactate (LAC) concentrations were measured before and immediately after the session, and 30 min after the session had ended. The LAC was determined by photometric reflectance on a validated Portable Accutrend Plus system (Roche, Sao Paulo, Brazil).

### Rating of Perceived Exertion (RPE)

RPE was collected as previously described (Tibana et al., 2018). The RPE was measured before, immediately after the exercise, and 30 min after the session, utilizing an adapted version of the Borg CR-10 scale (Foster et al., 2001; Morishita et al., 2018; Figure 2). The CR-10 scale is a 11-point Likert scale, varying from 0 to 10, with nominal descriptors attached to specific intensities. In order for participants to maintain the training intensity at the required effort (six out of 10), the RPE scale was explained to the participants individually, according to previous recommendations (Foster et al., 2001).

### Determination of the Internal Training Load

Edwards’ TRIMP (eTRIMP) was calculated based on the time spent in five predetermined training zones related to the participants’ maximal heart rate. The training zones and their weighting factor were as follows: zone 1 (50–59% HRmax—weighting factor = 1), zone 2 (60–69% HRmax—weighting factor = 2), zone 3 (70–79% HRmax—weighting factor = 3), zone 4 (80–89% HRmax—weighting factor = 4), and zone 5 (90–100% HRmax—weighting factor = 5). Each session’s eTRIMP was calculated by multiplying the time spent in each training zone by its weighing factor, and then summated to provide a total score.

bTRIMP was calculated based on training duration, changes in heart rate, and a weighting factor, according to the formula

$$\mathrm{TRIMP}=D\times \left(\Delta \;\mathrm{heart rate ratio}\right)\times e^{\left(b\;\;\times \;\;\Delta \;\mathrm{heart rate ratio}\right)},$$

where *D* = session duration, the constant *e* = 2.718, the weighting factor *b* = 1.67 for women and 1.92 for men, and ∆ heart rate ratio = (average heart rate − resting heart rate) ÷ (maximal heart rate − resting heart rate; Sanders et al., 2017).

The sRPE was calculated as the product of the session duration and the rate of perceived exertion 30 min after the session (RPE30; Foster, 1998; Derek et al., 2018; Tibana et al., 2018).

## Statistical Analysis

The data are expressed as mean value ± SD. Shapiro-Wilk test was used to check for normal distribution of the study variables (eTRIMP and bTRIMP, HR zones, and LAC presented a normal distribution). Non-parametric tests were used to analyze the RPE, as an ordinal scale with non-parametric distribution. A paired sample *t* test (and its correspondent for non-parametric data) was used to compare the LAC, bTRIMP and eTRIMP, HR zones, and RPE between ALL-OUT and RPE6 sessions of functional fitness. Cohen’s *d* was used to report effect sizes for the *t* tests (0.2: small, 0.5: medium, and 0.8: large), while effect sizes for non-parametric tests were calculated as *r* = *Z*/√*N* and interpreted as 0.10 (small), 0.30 (moderate), and 0.50 (large) effect (Glass and Hopkins, 1996). Chi-square for associations was used to explore the relationship between the percentage of time spent in each HR zone and each FFT session. The Spearman product moment correlation was used to assess the relationship between RPE and LAC, and RPE and HR, as well as the eTRIMP and bTRIMP relationship to RPE and LAC, with both conditions taken together for the two sessions. The magnitude of the correlations was classified as follows: *ρ* ≤ 0.1, trivial; 0.1 < *ρ* ≤ 0.3, small; 0.3 < *ρ* ≤ 0.5, moderate; 0.5 < *ρ* ≤ 0.7, large; 0.7 < *ρ* ≤ 0.9, very large; and *ρ* > 0.9, almost perfect (Glass and Hopkins, 1996).

## Results

Participants completed a higher number of repetitions (214.4 ± 18.6) during the ALL-OUT session when compared to the RPE6 session (190.5 ± 12.5). An in-depth discussion of these results and its implications has already been published (Tibana et al., 2019a).

The overall internal load and the physiological markers of strain for each session are presented in Table 2. When compared to the all-out session, blood lactate immediately after the session (18.9 ± 3.9 mmol L^−1^ vs. 12.8 ± 3.2 mmol L^−1^) and 30 min after the session (13.8 ± 3.5 mmol L^−1^ vs. 5.9 ± 1.6 mmol L^−1^) were significantly lower (*p* < 0.0005) in the RPE6 session. A significant difference between conditions was also found between the RPE immediately after the session (9.6 ± 0.7 vs. 6.2 ± 0.8; *p* = 0.011) and 30 min post session (3.9 ± 1.4 vs. 1.9 ± 0.7, *p* = 0.017), with the all-out session showing higher values.

**Table 2:** Differences in training loads, blood lactate concentration (LAC), and ratings of perceived exertion between the two functional fitness training (FFT) sessions.

	ALL-OUT	Rating of perceived exertion, RPE6	*p*	Effect size
Edward’s training impulse (eTRIMP), AU	93.1 ± 9.5	84.9 ± 13.7	0.085	0.70 (medium)
Bannister’s TRIMP (bTRIMP), AU	61.1 ± 10.6	55.7 ± 12.4	0.049	0.47 (small)
Session-rating of perceived exertion (sRPE), AU	91.7 ± 30.4	42.6 ± 14.9	0.002	1.82 (large)
LAC immediately after session, mmol/L	18.9 ± 3.9	12.8 ± 3.2	<0.0005	1.71 (large)
LAC 30 min after session, mmol/L	13.8 ± 3.5	5.9 ± 1.6	<0.0005	2.90 (large)
RPE immediately after session	9.6 ± 0.7	6.2 ± 0.8	0.011	4.52 (large)
RPE 30 min after session	3.9 ± 1.4	1.9 ± 0.7	0.017	1.81 (large)

**Figure 3:**
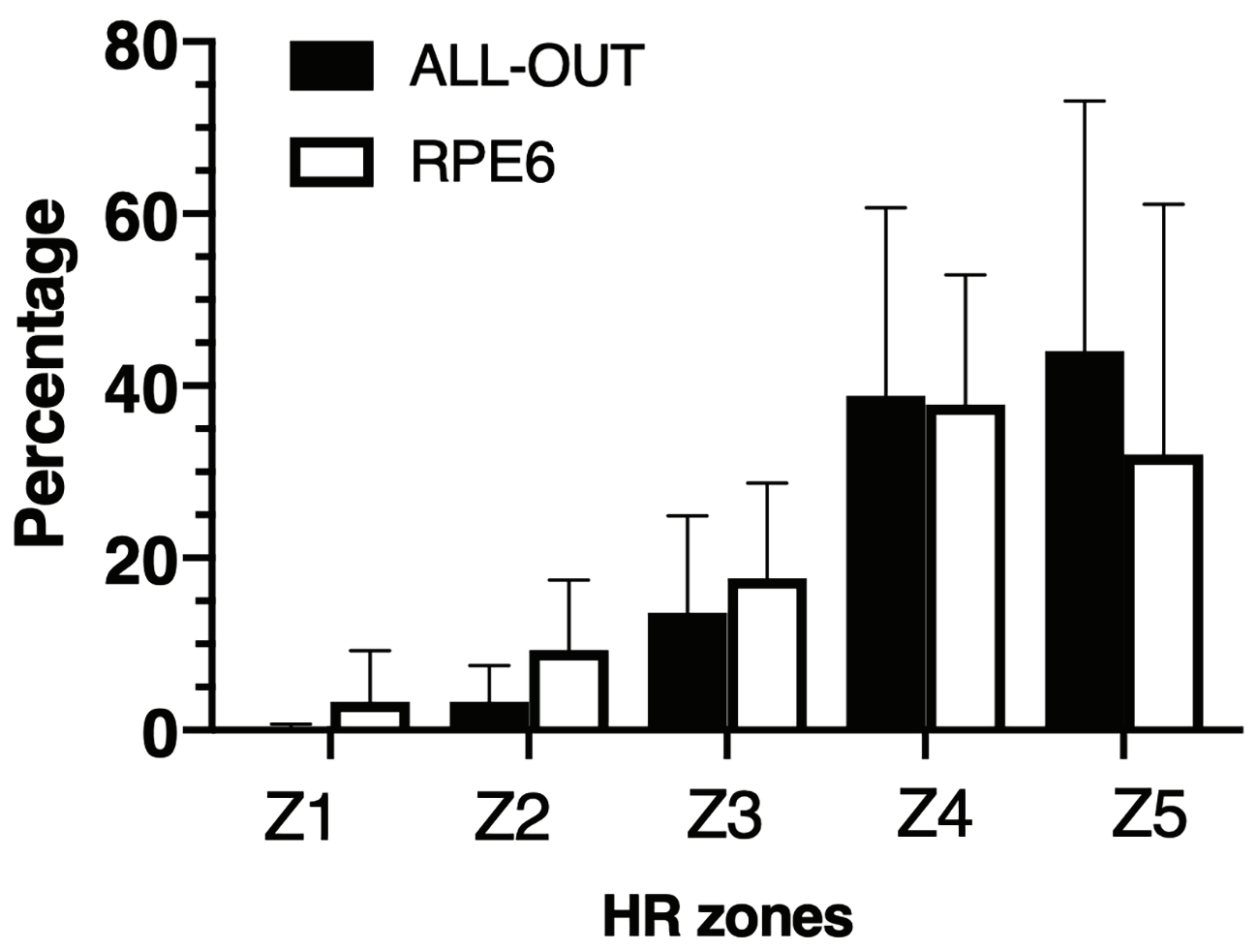
Percentage of time spent in each heart rate zone during the metabolic conditioning sessions.

A significant difference in the training load of the sessions was found for the bTRIMP (55.7 ± 12.4 AU for RPE6 vs. 61.1 ± 10.6 AU; *p* = 0.049) and sRPE (91.7 ± 30.4 AU vs. 42.6 ± 14.9 AU; *p* = 0.002), with the all-out session presenting higher values. No significant differences between the conditions were found when training loads were calculated using the eTRIMP method (93.1 ± 9.5 AU vs. 84.9 ± 13.7 AU; *p* = 0.085). There was no significant difference (*p* = 0.157) between ALL-OUT and RPE6 on the percentage of time spent in each HR zone (Figure 3).

**Figure 4:**
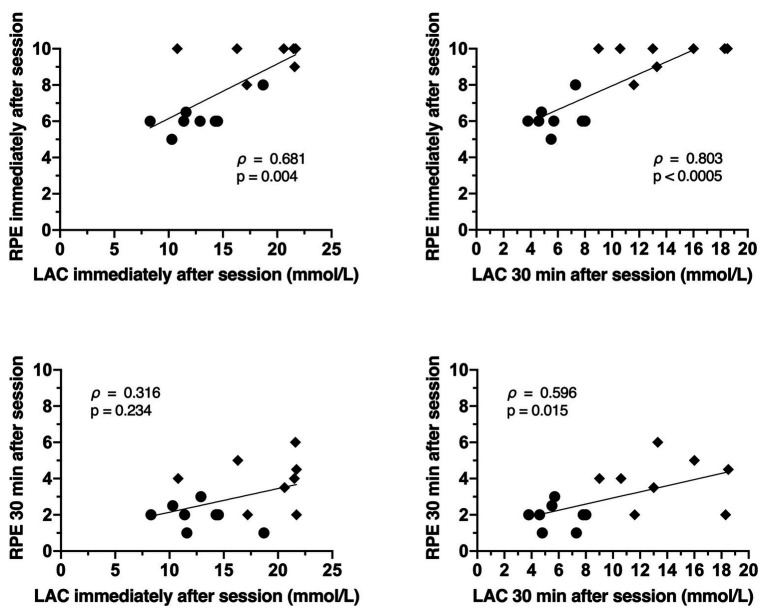
Correlations between rate of perceived exertion immediately after session **(top panels)** and blood lactate immediately and 30 min after session **(top panels)**, and rate of perceived exertion 30 min after the session and blood lactate immediately and 30 min after the session **(bottom panels)**.

**Figure 5:**
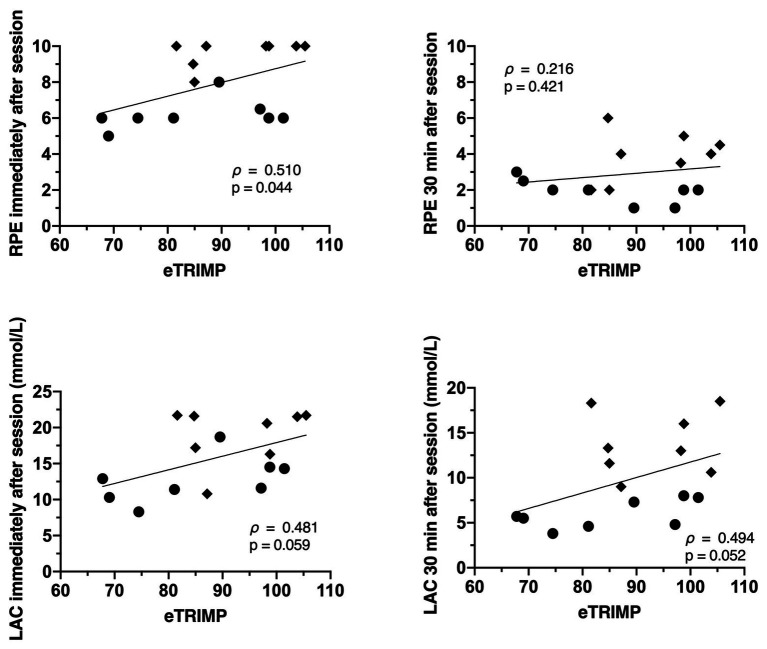
Correlation between eTRIMP and rate of perceived exertion immediately and 30 min after the conditioning session **(top panels)**, and between eTRIMP and blood lactate immediately and 30 min after the conditioning sessions **(bottom panels)**.

**Figure 6:**
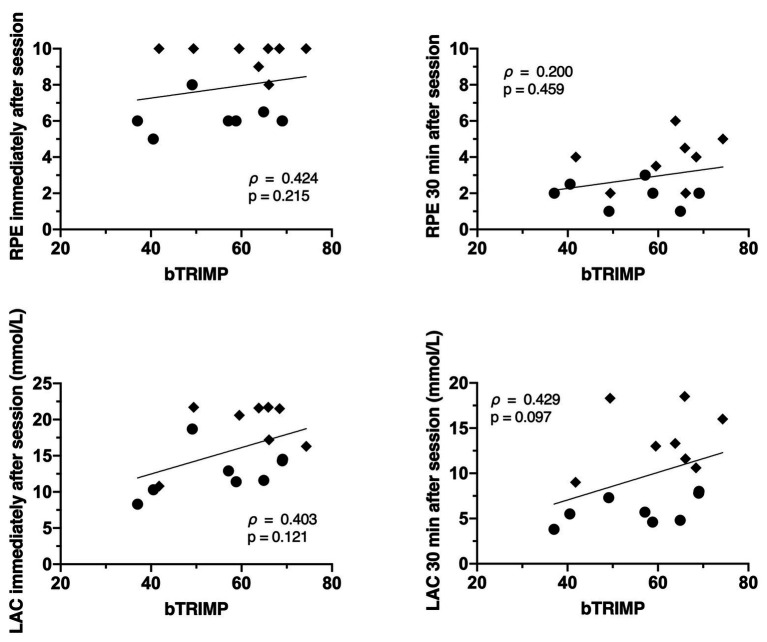
Correlation between bTRIMP and rate of perceived exertion immediately and 30 min after the conditioning session **(top panels)**, and between eTRIMP and blood lactate immediately and 30 min after the conditioning sessions **(bottom panels)**.

The relationships between the internal training loads (eTRIMP, bTRIMP, and sRPE) and physiological markers of strain are displayed in Figures 4–6. The RPE immediately post session was only significantly related to eTRIMP (*p* = 0.044; *ρ* = 0.510; large), while the RPE 30 min post session was not correlated to either eTRIMP (*p* = 0.421; *ρ* = 0.216) or bTRIMP (*p* = 0.200; *ρ* = 0.459). No significant correlations were observed between blood lactate concentration and eTRIMP or bTRIMP. RPE30 showed a significant correlation with lactate 30 min post session (*p* = 0.015; *ρ* = 0.596, large).

## Discussion

The results presented in this study only partially support our initial hypotheses. In particular, the use of eTRIMP was unable to distinguish between the overall training loads of the different sessions. The eTRIMP also showed no differences in the time spent at each training zone between the sessions, even though the all-out session had a greater amount of time spent at higher heart rate intensities (zones 4 and 5). The bTRIMP and sRPE were able to detect a significant difference in the overall training load of the sessions, with sRPE being more sensitive to such differences (*p* = 0.049, ES = 0.76 and *p* = 0.002, ES = 1.82, respectively). Both methods derived from HR (eTRIMP and bTRIMP) were not related to lactate immediately or 30 min post session. In contrast, sRPE showed a large correlation with blood lactate 30 min after the session (*ρ* = 0.596).

When comparing the three methods of internal load, our findings do not support previous research that found strong significant correlations between sRPE and eTRIMP (Derek et al., 2018; Tibana et al., 2018). One possible explanation is that the sessions in these studies had different durations (short vs. long), which can influence the magnitude of the correlations. Further, since our correlations spanned the different intensity conditions, it is possible that eTRIMP and bTRIMP might not provide an accurate assessment of internal training loads across an intensity range compared to sRPE. In addition, considering that the heart rate responses between conditions were similar regardless of the intensity of the sessions, this could also explain why eTRIMP and bTRIMP showed little or no differences between the training sessions.

This similar heart rate response can be explained by the nature of the exercises performed. During RT exercises, stroke volume seems to remain unchanged or is slightly decreased due to the mechanical occlusion caused by muscular contractions and to a high intramuscular pressure that can occlude blood flow to the working muscles (Lentini et al., 1993). Thus, to accommodate for the increased demand in cardiac output, an increase in heart rate is necessary (Beckham and Earnest, 2000). In addition, exercises that require stabilization of the core and/or significant coordination of upper and lower body, which can cause a natural Valsalva (breath holds) maneuver, can also lead to an increase in heart rate. Therefore, the oxygen demand by the working muscles along with possible additional heart rate responses from breath holds and changes in thoracic pressure can lead to an overestimated heart rate response even when the intensity of the session is controlled (such as in our RPE6 condition). As previous studies only included sessions performed at an all-out intensity (Derek et al., 2018; Tibana et al., 2018), it is possible that when the intensity is lower (controlled by RPE), methods based on heart rate do not provide an accurate assessment of the training loads of sessions performed at different intensities.

Therefore, similar to what occurs with traditional resistance training (RT; Scott et al., 2016), it seems that heart rate-based methods might not be the most accurate to monitor training loads in FFT. In this context, finding a measure of internal training load that is practical, reliable, and user-friendly is a challenge in FFT. It has been suggested that a valid measure of training load should show a strong dose-response relationship with a particular training outcome, such as fitness level, fatigue status, or injury risk (Halson, 2014; Bourdon et al., 2017). However, many of the commonly used markers to monitor internal training loads present additional challenges related to their ease of use, validity, and reliability (Halson, 2014; Bourdon et al., 2017).

For example, questionnaires and heart rate variability have not been shown to be consistently related to changes in training loads during FFT sessions and programs (Drake et al., 2017; Allyson et al., 2018; Tibana et al., 2019b). Changes in hormonal concentrations, such as testosterone and cortisol (Mangine et al., 2018), and biochemical markers, such as serum creatine kinase (Timón et al., 2019), have been shown during periods of increased training and competition stress in FFT. While the use of these has been recommended as a monitoring strategy (Mangine et al., 2018; Timón et al., 2019), such markers are often costly, time-consuming, and difficult to be applied in a practical setting (Halson, 2014). In addition, such measures of internal training load have been challenged, as these are considered a measure of the post-exercise response to the prescribed internal training load, rather than a marker of the internal training load *per se* (Impellizzeri et al., 2019).

Given the challenges associated with monitoring training loads in FFT, it seems that sRPE provides the best option. In sports that face similar challenges when it comes to monitoring training loads, such as taekwondo and water polo (Lupo et al., 2014, 2017), sRPE seems to be more exhaustive in evaluating different aspects regarding the internal training load of the sessions. In addition, previous studies have shown that sRPE is a reliable, low-cost method (Derek et al., 2018; Tibana et al., 2018) that is correlated to physiological stress and workload following FFT sessions. This was also the case in this study. The use of sRPE was able to provide accurate training loads that were significantly different between the conditions. The RPE 30 min after the sessions (used to calculate sRPE) was also strongly correlated to blood lactate 30 min post exercise, indicating that when the sRPE measure is taken, the physiological status of the participant is considered in the evaluation of the FFT session.

However, caution is advised when assessing the RPE-lactate relationship as it has been shown to be altered during prolonged sessions (Green et al., 2005) and during bouts of repeated exercise (Fusco et al., 2020a), situations that can occur during FFT sessions. Still, the relationship might also warrant further investigation in FFT as previous studies have demonstrated that it can be a surrogate for monitoring accumulated fatigue over a training period (Halson, 2014). Nevertheless, as the metabolic conditioning sessions that occur within a FFT program are seldom repeated, the use of the RPE-lactate relationship would require an “index workout” to be established. This would require a specific session to be repeated at regular intervals, potentially reducing the practical applicability of this tool to FFT.

Despite promising results, further investigations on the use of sRPE in long-term monitoring are needed. Specifically, long-term studies that can assess the efficacy of the method in detecting changes in participant’s fitness levels, performance, and fatigue status are necessary. In addition, a recent study (Fusco et al., 2020b) demonstrated that sRPE may significantly increase as training loads progress, supporting the concept that sRPE might reflect information beyond the internal intensity of exercise (reflecting accumulating fatigue in addition to exercise intensity). It has also been shown that sRPE may provide information about accumulated fatigue during a single prolonged training bout (Fusco et al., 2020a), while other markers of intensity, such as HR and lactate concentration, might not change. Therefore, sRPE might be a sensitive tool for monitoring the internal training load that also provides further information on accumulated fatigue. As previously mentioned, potential markers that could reflect this fatigue in FFT are either impractical or have not shown consistent results. Caution is advised, however, as previous research has shown that sRPE might still be prone to training load errors in FFT (Derek et al., 2018), particularly in the early stages of its utilization. In addition, a case study has shown no correlation between internal training load measured with sRPE and heart rate variability or the participant’s subjective recovery based on wellness questionnaires (Tibana et al., 2019b). Therefore, it is possible that a combination with another metric might be necessary to ensure accurate measurement of training loads in FFT.

## Conclusion

The results show that bTRIMP and sRPE were able to detect a significant difference in the training loads between sessions, with no changes detected with eTRIMP. Only sRPE was related to the physiological strain of the sessions. While bTRIMP could be used to monitor the intensity of FFT sessions, it seems that the method is not the most accurate option when sessions are performed at different intensities. In addition, the use of heart rate monitors during FFT sessions could present a challenge given the nature of some exercises. The cost and the knowledge required to analyze heart rate data from the session might also present a barrier to its use. In this context, sRPE seems to be more advantageous given its ease of use and applicability to the types of sessions that are performed in FFT. As practitioners are advised to use methods that are simple, time‐ and cost-effective (Coutts, 2014), the use of the sRPE method is currently recommended to monitor training loads in FFT.

## Data Availability Statement

The datasets generated for this study are available on request to the corresponding author.

## Ethics Statement

The studies involving human participants were reviewed and approved by the Research Ethics Committee for Human Use (2.698.225/Universidade Estácio de Sá/UNESA/RJ) and conformed to the Declaration of Helsinki on the use of human participants for research. The patients/participants provided their written informed consent to participate in this study.

## Author Contributions

RT and FV contributed to the conception and design of the study. RT and NS contributed to statistical analysis. JN wrote the first draft of the manuscript and was responsible for further edits. JP, RT, MK, NS, and FV wrote and edited sections of the paper. All authors contributed to manuscript revisions and have read and approved the submitted version.

### Conflict of Interest

The authors declare that the research was conducted in the absence of any commercial or financial relationships that could be construed as a potential conflict of interest.
